# The Uncounted Casualties of a Hidden COVID-19 Epidemic in China: Cross-sectional Study on Deaths Related to Overwork

**DOI:** 10.2196/23311

**Published:** 2021-04-20

**Authors:** Zhicheng Wang, Leesa Lin, Yan Guo, Huayi Xiong, Kun Tang

**Affiliations:** 1 Vanke School of Public Health Tsinghua University Beijing China; 2 School of Medicine Tsinghua University Beijing China; 3 Department of Infectious Disease Epidemiology London School of Hygiene & Tropical Medicine London United Kingdom; 4 Department of Health Policy and Management Peking University Beijing China; 5 School of Health Humanities Peking University Beijing China

**Keywords:** nonpharmaceutical interventions, on-duty deaths, COVID-19, overwork death, crowdsourced data, intervention, mortality, casualty, cross-sectional, overwork, stress

## Abstract

**Background:**

During the COVID-19 response, nonclinical essential workers usually worked overtime and experienced significant work stress, which subsequently increased their risk of mortality due to cardiovascular diseases, stroke, and pre-existing conditions. Deaths on duty, including deaths due to overwork, during the COVID-19 response were usually reported on web-based platforms for public recognition and solidarity. Although no official statistics are available for these casualties, a list of on-duty deaths has been made publicly available on the web by crowdsourcing.

**Objective:**

This study aims to understand the trends and characteristics of deaths related to overwork among the frontline nonclinical essential workers participating in nonpharmaceutical interventions during the first wave of COVID-19 in China.

**Methods:**

Based on a web-based crowdsourced list of deaths on duty during the first wave of the COVID-19 response in China, we manually verified all overwork-related death records against the full-text web reports from credible sources. After excluding deaths caused by COVID-19 infection and accidents, a total of 340 deaths related to overwork among nonclinical essential workers were attributed to combatting the COVID-19 crisis. We coded the key characteristics of the deceased workers, including sex, age at death, location, causes of death, date of incidence, date of death, containment duties, working area, and occupation. The temporal and spatial correlations between deaths from overwork and COVID-19 cases in China were also examined using Pearson correlation coefficient.

**Results:**

From January 20 to April 26, 2020, at least 340 nonclinical frontline workers in China were reported to have died as a result of overwork while combatting COVID-19. The weekly overwork mortality was positively correlated with weekly COVID-19 cases (*r*=0.79, *P*<.001). Two-thirds of deceased workers (230/340, 67.6%) were under 55 years old, and two major causes of deaths related to overwork were cardiovascular diseases (138/340, 40.6%) and cerebrovascular diseases (73/340, 21.5%). Outside of Hubei province, there were almost 2.5 times as many deaths caused by COVID-19–related overwork (308/340, 90.6%) than by COVID-19 itself (n=120).

**Conclusions:**

The high number of deaths related to overwork among nonclinical essential workers at the frontline of the COVID-19 epidemic is alarming. Policies for occupational health protection against work hazards should therefore be prioritized and enforced.

## Introduction

The first wave of the COVID-19 epidemic in China was brought under control within 3 months—from mid-January 2020 (when human-to-human transmission of COVID-19 was confirmed) to the end of April 2020 [1]. The effective containment of the initial wave of COVID-19 in China was credited to not only the frontline medical response but also swift, massive, and aggressive nonpharmaceutical interventions (NPIs) [2]. Implementation of such interventions was time-sensitive and labor-intensive, demanding the continuous efforts of staff from all sectors to meet community health care and logistical needs, such as setting up checkpoints for temperature screening, conducting travel history inquiries to screen for suspected cases, and protecting incarcerated people in prisons and detention houses. However, although COVID-19 cases and related deaths among medical professionals have been well acknowledged [3], the health of nonclinical essential workers engaged in NPIs to contain the spread of COVID-19 should not be overlooked.

In China, these frontline workers have suffered from high levels of psychological and physical stress, and they have worked long hours without sufficient rest for weeks, even months, due to the rapid acceleration of the epidemic and understaffing [4]. It has long been recognized that “overwork” can kill [5], as prolonged working hours and heightened psychological stress increase the risk of coronary heart disease and stroke by inducing increased catecholamine secretion, eventually leading to increased mortality risk [6-8]. However, the hidden casualties of these deaths from overwork have not been studied. Deaths on duty during the COVID-19 response were usually reported by web-based news platforms for reasons of public recognition and solidarity, but there are no official statistics concerning these casualties. Fortunately, a list of on-duty deaths in the combat against the COVID-19 crisis has been made publicly available on the social media platform Weibo [9] by crowdsourcing and has been recognized by the Chinese public. This study aims to conduct a comprehensive search of web-based news reports to describe the trends and characteristics of deaths from overwork in the fight against the COVID-19 epidemic in China.

## Methods

Based on a widely recognized list of on-duty deaths in the fight against COVID-19 in China, from January 20 to April 26, 2020, a total of 496 deaths on duty were included in this study, with no duplication [9]. All records of deaths in the line of duty were verified with the corresponding full-text, reports on the web from credible news platforms such as Xinhua News Agency, People’s Daily, Sohu.com, and Sina.com, as well as official government websites (detailed sources are presented in Multimedia Appendix 1). If the on-duty death reports explicitly mentioned *overwork* and the deaths were not attributed to COVID-19 or accidents, then those deaths would be identified as *deaths related to overworks*. After excluding the deaths caused by COVID-19 or accidents, we found that 340 of the 496 (68.5%) deaths related to overwork among nonclinical essential workers were attributed to combatting the COVID-19 crisis.

We coded the key characteristics of the 340 deceased workers, including sex, age at death, location, causes of death, date of incidence, date of death, containment duties, working area, and occupation (see Table S1 in Multimedia Appendix 1). The date of incidence refers to the date when the frontline worker’s health condition deteriorated suddenly due to overwork, and they could no longer perform their duties, whereas the date of death refers to the date when the overwork-related death occurred. If a frontline worker died from overwork immediately or was found dead, the date of incidence and the date of death would be the same. However, if the frontline worker’s health condition deteriorated suddenly due to overwork, but they died only after several days of rescue and treatment at the hospital, then the date of incidence and date of death would be different. This study only used the date of incidence to analyze the trends and correlation of deaths from overwork and COVID-19 incidence, as it reflected the timely physical and psychological stress caused by the severity of the epidemic.

In addition, the number of daily deaths from overwork and daily COVID-19 incidences were aggregated into weekly COVID-19 incidence to reflect the time trends of deaths from overwork and COVID-19 incidence [10]. The temporal correlation between weekly overwork mortality and COVID-19 incidence and the spatial correlation between provincial deaths from overwork and COVID-19 case counts were also examined using Pearson correlation coefficient (*r*); the correlation was considered statistically significant at *P*<.05. As a sensitivity analysis, we analyzed cases in which the death occurred within 2 days of the date of incidence to examine the correlation. This study was approved by the institutional review board of Research Center for Public Health, School of Medicine, Tsinghua University, Beijing, China (THUSM/PHREC 2020400-009).

## Results

**Figure 1:**
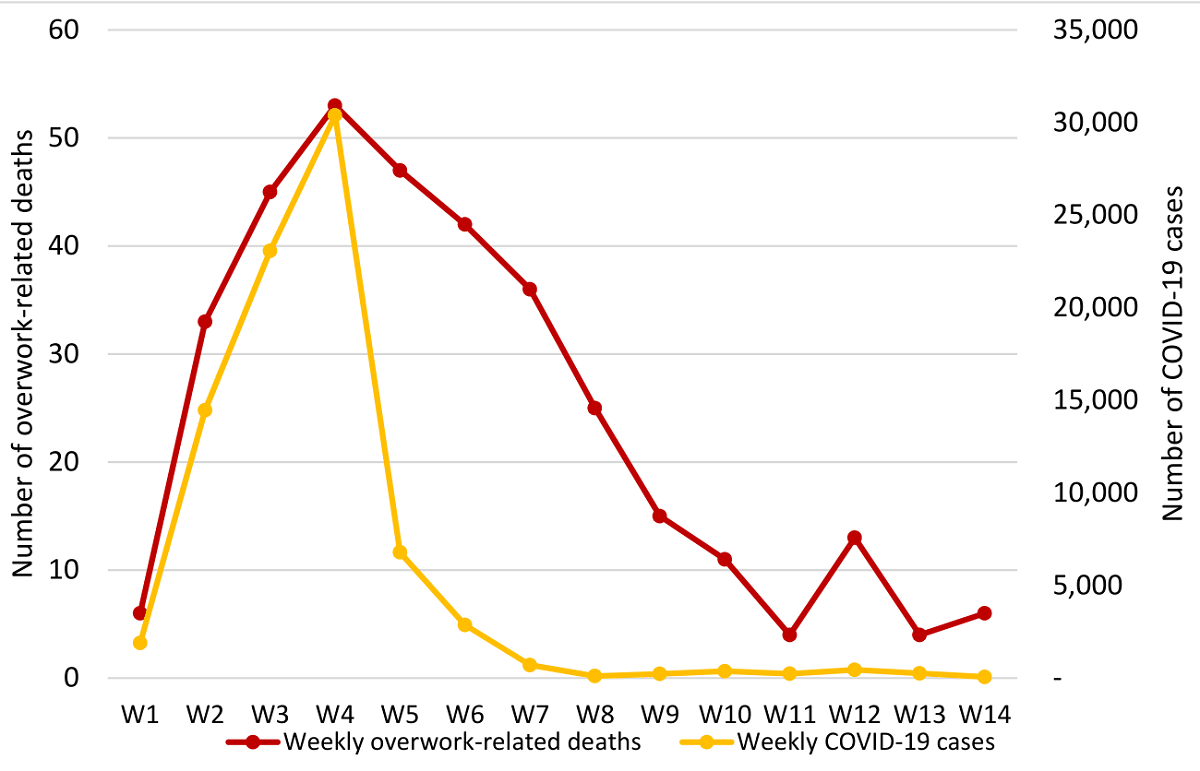
Trends of weekly overwork-related deaths and COVID-19 incidence between January 20 and April 26, 2020, in China.

The earliest deaths from overwork occurred on January 24, 2020, and during the 14 weeks from January 20 to April 26, 2020, at least 340 nonclinical frontline workers in China were reported to have died due to overwork in the fight against the COVID-19 epidemic. Both the number of COVID-19 cases (n=30,396) and the number of deaths from overwork (n=53) reached the peak in the fourth week after January 20 (Figure 1). Weekly overwork mortality was positively correlated with weekly COVID-19 cases (*r*=0.79, *P*<.001). A total of 86.5% (294/340) of cases died within 2 days of the incidence. As indicated by the sensitivity analysis, if only considering the deaths within 2 days of incidence, the temporal correlation between weekly overwork mortality and weekly COVID-19 cases would increase to 0.81 (*P*<.001).

Among those individuals who died due to overwork (Table 1), the mean age at death was 49.50 (SD 9.13) years, with two-thirds (230/340, 67.6%) of them under 55 years old. Two of the major specified underlying causes of death were cardiovascular diseases (138/340, 40.6%), including myocardial infarction and sudden cardiac arrest, and cerebrovascular diseases (73/340, 21.5%), including stroke. A majority of the deaths from overwork were among men (324/340, 95.3%) in China; this reflected that the epidemic containment responsibilities were carried out by village leaders (111/340, 32.6%), police (96/340, 28.2%), and civil servants (58/340, 17.1%), which are traditionally male-dominated occupations in China. Most deceased workers were involved in community mobilization and support (217/340, 63.8%), including community closure and access control, temperature screening, travel history collection, and delivery of daily necessities, followed by public security (77/340, 22.6%). Apart from working in rural (156/340, 45.9%) and urban (132/340, 38.8%) settings, 15.3% (52/340) of the deceased workers used to work at traffic checkpoints on expressways and in prisons and detention houses.

In addition, 32 of the 340 (9.4%) deaths from overwork occurred in Hubei province, which reported the highest prevalence of COVID-19 in China. Among the remaining 308 (90.6%) deaths reported outside Hubei province, provincial deaths related to overwork were positively correlated with the number of COVID-19 cases (*r*=0.55, *P*=.002). If only considering the deaths that occurred within 2 days of incidence, the correlation coefficient remains nearly the same (*r*=0.55, *P*=.002).

**Table 1:** Characteristics of documented frontline workers who died due to overwork during the emergency response to COVID-19 in China between January 20 and April 26, 2020 (N=340).

Characteristic			Value, n (%)
**Sex**			
	Female	16 (4.7)	
	Male	324 (95.3)	
**Age range (years)**			
	25-34	21 (6.2)	
	35-44	69 (20.3)	
	45-54	140 (41.2)	
	55-64	92 (27.1)	
	65-71	15 (4.4)	
	Unspecified	3 (0.9)	
**Working area**			
	Rural	156 (45.9)	
	Urban	132 (38.8)	
	Other^a^	52 (15.3)	
**Underlying cause of death**			
	Cardiovascular diseases	138 (40.6)	
	Cerebrovascular diseases	73 (21.5)	
	Other specific causes^b^	27 (7.9)	
	Unspecified	102 (30)	
**Epidemic containment duty**			
	Community mobilization and support	217 (63.8)	
	Public security	77 (22.6)	
	Traffic checkpoint and control	30 (8.5)	
	Other^c^	17 (5)	
**Occupation**			
	Village leaders	111 (32.6)	
	Police	96 (28.2)	
	Civil servant	58 (17.1)	
	Volunteer	32 (9.4)	
	Other public sectors^d^	26 (7.1)	
	Corporate employee	17 (5)	
**Location**			
	Hubei province	32 (9.4)	
	Outside Hubei province	308 (90.6)	
**Difference between date of incidence and data of death (days)**			
	0	244 (71.8)	
	1	36 (10.6)	
	2	14 (4.1)	
	>2	46 (13.5)	

^a^Other working areas include prisons, detention houses, and traffic checkpoints at the expressway.

^b^Other causes of death include acute hepatic failure, acute pancreatitis, and pre-existing conditions.

^c^Other epidemic containment duties include logistics, electricity or telecommunication maintenance, and health communication.

^d^Other public sectors include schools and institutions funded by the government.

## Discussion

### Principal Findings

This study utilized the manually verified crowdsourced data available on the internet to illustrate the trends and characteristics of deaths from overwork, which were casualties of the effort to contain the COVID-19 epidemic in China. The 340 deaths from overwork reveal a hidden “epidemic within the epidemic” that has not been documented thus far and has further sounded the alarm of fatigue and occupational burnout among nonclinical frontline workers. The severity of the epidemic presented a considerable health burden to those who were involved in the battle against COVID-19, and it explains why the peak of deaths from overwork was synchronized with the peak of the COVID-19 pandemic. Notably, outside Hubei province, there were over 2.5 times as many deaths caused by COVID-19–related overwork (308/340, 90.6%) than deaths caused by the disease itself (n=120) [10], and these provincial deaths related to overwork were correlated with COVID-19 case counts, signaling the high intensity of NPIs to curb the COVID-19 epidemic.

This epidemic embodies the health threats that all nonclinical essential workers faced. First, nonclinical essential workers had mental distress due to the fear of contracting COVID-19. Unlike ordinary residents under home quarantine, nonclinical essential workers had to work outside to perform their duties, especially screening for suspected COVID-19 cases. During the early stages of the epidemic in China, all personal protective equipment (PPE) was prioritized for clinical staff [11]; thus, nonclinical workers faced a shortage of PPE, which may have heightened their fears of COVID-19 infection. Second, most of the containment strategies were implemented by public sector employees, and they were accountable by the law and regulation for containing the epidemic. Thus, they experienced considerable work stress and workload due to understaffing, especially in provinces other than Hubei. The Chinese central government dispatched many clinical and nonclinical workers from other provinces to support Hubei province [12], whereas for other provinces, they had to manage to contain the transmission of COVID-19 largely on their own. Nonclinical essential workers worked overtime at the peak of the epidemic, which may have increased the risk of death due to cardiovascular and cerebrovascular diseases [6,7]. Third, due to understaffing, many workers with pre-existing conditions joined the workforce in the intensive battle against COVID-19. Those workers did not have enough time to rest and recover, and their pre-existing poor health conditions may have deteriorated and contributed to the observed deaths from overwork.

The high number of deaths from overwork among essential workers serving at the frontline during the COVID-19 epidemic is alarming. Although protection for health care workers in the line of duty against COVID-19 has been a global focus [11,13], policies for occupational health protection against work hazards such as adequate training, sufficient PPE, and proper working shifts, should be prioritized and enforced. Counseling services should also be provided to mitigate the psychosocial impacts. The possibility of a second wave of COVID-19 cannot be ruled out [14], so NPIs will likely remain in place until there is a breakthrough in treatment or a vaccination becomes widely available.

### Limitations

Our study also has some limitations. First, the deaths related to overwork were identified based on news reports on the web, not from the hospital health records; hence, some misclassification of data may exist. We verified the full-text reports of all deaths from overwork by using credible and official sources to minimize this misclassification. Second, the length and details of the individual death reports varied and, as such, some characteristics could not be retrieved and coded. Finally, the number of deaths from overwork was likely to be underestimated, as not every incident was reported on the internet.

### Conclusions

This cross-sectional study based on web-based crowdsourced data emphasizes that deaths related to overwork among nonclinical essential workers should be acknowledged. Corresponding policies for occupational health protection against deaths due to overwork should be implemented. Nonclinical essential workers are the unsung heroes, and their safety and well-being should be prioritized as they constitute society’s frontline of defense against COVID-19.

## Appendix

Multimedia Appendix 1 Table S1. Deidentified individual characteristics of documented frontline workers who died due to overwork during the emergency response to COVID-19 between January 20 and April 26, 2020, in China.

## Abbreviations

NPIs: nonpharmaceutical interventions
PPE: personal protective equipment
